# Cobalt-Immobilized Microplastics as a Functional Catalyst for PMS-Based Nitrate Degradation: Optimization Using Response Surface Methodology

**DOI:** 10.3390/molecules30234591

**Published:** 2025-11-29

**Authors:** Mohammad Javad Amiri, Mehdi Bahrami, Anahita Zare, Mohammad Gheibi

**Affiliations:** 1 Department of Water Science and Engineering, Faculty of Agriculture, Fasa University, Fasa 74616-86131, Iran; bahrami@fasau.ac.ir (M.B.);; 2 Research Institute of Water Resources Management in Arid Region, Fasa University, Fasa 74616-86131, Iran; 3 Institute for Nanomaterials, Advanced Technologies and Innovation, Technical University of Liberec, Studentská 1402/2, 461 17 Liberec, Czech Republic

**Keywords:** water treatment, cobalt catalyst, peroxymonosulfate activation, nitrate degradation, process optimization

## Abstract

Nitrate contamination of water resources poses significant ecological and public health risks. This study developed a cobalt-immobilized microplastic catalyst (Co–MP) capable of activating peroxymonosulfate (PMS) and facilitating formic-acid-assisted catalytic denitrification of nitrate. Characterization via Scanning Electron Microscopy (SEM), Fourier Transform Infrared Spectroscopy (FTIR), Energy-Dispersive X-ray Spectroscopy (EDX), and X-ray diffractometry (XRD) confirmed successful Co deposition, with the surface cobalt content reaching 5.2%. The system’s performance was optimized using Response Surface Methodology (RSM), identifying catalyst dosage and Co(II) concentration as the most significant factors. Under the optimized conditions (pH 5.5, reaction time 120 min, catalyst dosage 1.5 g L^−1^, and Co(II) concentration 60 mg L^−1^), the system achieved a nitrate removal efficiency of 90.6%, in excellent agreement with the model prediction (90.93%), along with an 86.7% reduction in total nitrogen, confirming stepwise denitrification to gaseous nitrogen species (N_2_). The Co(II)/Co(III) redox cycle, sustained by PMS-assisted regeneration and driven by formic acid as the electron donor, ensured stable performance with minimal cobalt leaching (0.05 mg L^−1^). This coupled oxidative–reductive system offers a sustainable dual-remediation strategy that simultaneously achieves selective nitrate conversion and valorizes microplastic waste for catalytic environmental applications.

## 1. Introduction

Nitrate contamination of water resources has been recognized as a critical environmental issue worldwide, particularly in Iran [1], due to intensive agricultural practices, industrial effluents, and urban runoff [2,3]. Excessive use of nitrogen-based fertilizers and the discharge of untreated wastewater have led to elevated nitrate levels in surface and groundwater, posing severe ecological and public health risks [2,3]. Prolonged exposure to nitrate-contaminated drinking water has been linked to methemoglobinemia (“blue baby syndrome”), thyroid disorders, and potential carcinogenic effects through the formation of N-nitroso compounds [2,3]. In this regard, the World Health Organization (WHO) suggests that consuming water with nitrate levels exceeding 10 mg NO_3_^−^-N L^−1^ (or 50 mg L^−1^ as nitrate) may increase health risks [4]. Consequently, the development of efficient and sustainable strategies for nitrate removal or transformation has become an urgent priority in water treatment research.

Among various treatment techniques, advanced oxidation processes (AOPs) have gained considerable attention due to their ability to generate highly reactive oxygen species (ROS), such as hydroxyl radicals (^•^OH) and sulfate radicals (${SO_{4}^{\cdot }}^{-}$), capable of degrading a wide range of organic pollutants [5,6]. Compared with conventional treatments, AOPs are particularly attractive for water purification since they allow rapid reaction kinetics, strong oxidative potential, and applicability under diverse water matrices [7]. In recent years, peroxymonosulfate (PMS) activation has emerged as an efficient AOP pathway, offering advantages such as controlled radical generation and strong stability across different pH conditions [5,6,8,9,10]. Transition metals, particularly cobalt, are widely recognized for their superior catalytic performance in PMS activation due to their redox cycling ability between Co(II)/Co(III), which facilitates continuous radical generation [5,11]. However, a persistent challenge in PMS-based systems is the immobilization and recovery of cobalt catalysts to prevent secondary contamination [5,12]. To address this, innovative approaches have explored the use of solid supports for cobalt stabilization [5,13].

Microplastics (MPs), defined as synthetic solid particles or polymeric matrices with dimensions less than 5 mm, are now recognized as a pervasive class of environmental contaminants. Their physicochemical properties, such as size, density, surface chemistry, and hydrophobicity, govern their environmental fate and interactions with other contaminants [14,15]. Derived primarily from non-renewable resources, MPs are inexpensive and widely available synthetic polymers that persist in natural systems due to their inherent resistance to degradation [14,15]. Beyond their role as direct environmental pollutants, MPs exhibit a high sorption capacity for co-occurring contaminants. This property enables MPs to act as vectors, concentrating and transporting harmful substances such as heavy metals and persistent organic pollutants through aquatic systems [16]. This dual nature has inspired research into repurposing MPs as functional substrates in water treatment, where their durability and pollutant-binding capacity can be leveraged to immobilize catalysts or act as carriers for pollutant removal. Thus, despite their environmental drawbacks, MPs hold potential as low-cost supports in advanced remediation technologies.

To systematically optimize the process, Response Surface Methodology (RSM) was employed to evaluate the influence and interactions of key operational parameters on nitrate removal efficiency. By using a statistically designed set of experiments, RSM minimizes the total number of experimental runs required, thereby reducing time, energy consumption, and costs, while still ensuring accurate determination of optimal conditions [17,18]. This approach not only advances the mechanistic understanding of PMS-based AOPs for nitrate remediation but also underscores the innovative application of MPs as functional supports for cobalt immobilization in environmental catalysis.

Despite extensive research on PMS activation systems and cobalt-based catalysts for the degradation of organic contaminants [19,20,21,22], critical knowledge gaps remain regarding their application to inorganic nitrogen pollution, particularly nitrate. First, it is still unclear whether and how Co-activated PMS systems can mediate nitrate transformation, given that PMS activation is traditionally associated with oxidative pathways and not with multi-electron reduction processes relevant to denitrification. Second, although MPs represent an abundant and low-cost substrate, strategies for their robust functionalization as safe catalytic supports—capable of anchoring cobalt with minimal leaching under reaction conditions—remain underdeveloped. Third, an integrated understanding is lacking on how oxidant dosage, sacrificial electron donors, and operational parameters (pH, reaction time, cobalt availability) can be tuned cooperatively to promote selective, multi-electron nitrate reduction pathways rather than undesirable side reactions. Addressing these gaps is essential for enabling PMS-based denitrification systems that are both mechanistically sound and environmentally practical. Therefore, in this study, a Co–MP system was developed as a novel catalyst for the activation of peroxymonosulfate (PMS) toward nitrate transformation. The work aimed to evaluate the dual functionality of MPs as both pollutant repurposing carriers and stabilizers for cobalt, thereby enhancing catalytic activity and reducing metal leaching. The synthesized catalyst was comprehensively characterized using Fourier Transform Infrared Spectroscopy (FTIR), Scanning Electron Microscopy (SEM), Energy Dispersive X-ray Spectroscopy (EDAX), X-ray diffractometry (XRD) and Brunauer–Emmett–Teller (BET) surface analysis to elucidate its physicochemical properties. Nitrate degradation performance was assessed using ion chromatography (IC) to monitor residual anions and cations, providing insight into the efficiency and selectivity of the process.

## 2. Results and Discussion

### 2.1. Characterization of the Co–MP Catalyst

**Figure 1:**
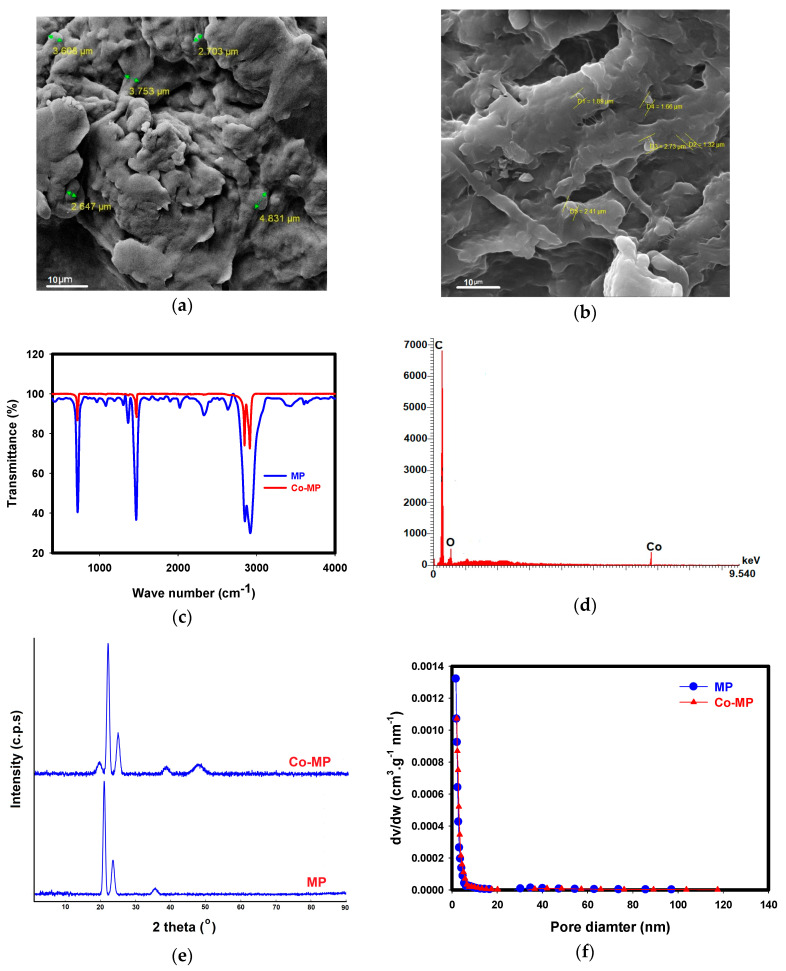
SEM image of MP (**a**); SEM image of Co–MP (**b**); FTIR spectra of MP and cobalt-immobilized MP (**c**); EDX spectrum of Co–MP (**d**); XRD patterns of MP and Co-MP (**e**); Pore size distribution of MP and Co-MP (**f**).

The physicochemical properties of the synthesized Co–MP catalyst were analyzed to confirm the successful immobilization of cobalt species on the MP surface and to understand their potential influence on catalytic performance. The untreated MP surface is irregular and coarse, with rough flakes and deep cavities typical of weathered polymer debris [15] (Figure 1a), while the Co–MP MP at 10 µm magnification shows a smoother texture covered by fine granular deposits (Figure 1b). The cobalt treatment clearly modifies the surface, forming small, dense clusters that partially fill pores and coat the polymer. This coating gives the material a more compact and uniform appearance compared to the jagged, fractured morphology of the original MP. The contrast difference suggests the presence of cobalt particles, and the overall reduction in pore depth indicates successful immobilization. Together, these observations confirm that cobalt deposition significantly alters the MP’s surface structure, producing a more functional and reactive material. The FTIR spectra of the MP and Co-MP clearly demonstrate chemical modifications that confirm successful cobalt immobilization (Figure 1c). The FTIR spectrum of pristine MP shows a broad band near ~3400 cm^−1^, which is attributed to O–H stretching vibrations originating primarily from surface-adsorbed moisture. After cobalt immobilization, this O–H band is no longer observed, indicating that cobalt binding either consumes or masks these surface hydroxyl sites, resulting in a cleaner spectrum without the broad O–H feature. In the MP spectrum, distinct absorption bands appear around 2915–2845 cm^−1^ (C–H stretching of aliphatic chains), 1460 cm^−1^ (C–H bending), and 720 cm^−1^ (rocking vibration of –(CH_2_)n–), all characteristic of polyethylene-based MP [15,23]. After cobalt immobilization, the Co-MP spectrum retains these fundamental polymer peaks but shows noticeable changes in band intensity and slight shifts in several regions. A small reduction in transmittance and minor peak shifts near 1720–1650 cm^−1^ suggest the formation or enhancement of carbonyl and hydroxyl groups, which likely originate from surface oxidation and act as anchoring sites for cobalt ions. The reduced peak intensity in the C–H stretching region and subtle broadening around 3400 cm^−1^ indicate possible coordination between Co species and surface oxygenated functional groups (–OH, –C=O). These changes imply that cobalt ions interacted chemically rather than merely depositing physically.

The EDX results strongly support and align with the observations from both SEM and FTIR analyses (Figure 1d). In the untreated MP, the surface is predominantly composed of carbon (96.1%) with a minor oxygen content (3.9%), typical of hydrocarbon-based polymers. After cobalt immobilization, the carbon percentage decreases to 88.2%, while oxygen increases to 6.6% and cobalt appears at 5.2%. This shift clearly indicates successful deposition of cobalt species on the polymer surface along with surface oxidation. The increase in oxygen content suggests the formation of oxygen-containing functional groups, such as hydroxyl and carbonyl, which serve as active binding sites for cobalt ions. These chemical modifications are consistent with the FTIR results, where new or shifted absorption peaks associated with –OH and C=O groups were observed, confirming interaction between cobalt and the oxidized polymer surface. Similarly, SEM images showed the appearance of fine granular deposits and smoother coated regions compared to the rough texture of the pristine microplastic, indicating cobalt particle attachment. Therefore, the EDX data, together with SEM morphology and FTIR spectral changes, consistently demonstrate that cobalt was successfully immobilized onto the MP surface through chemical interactions involving oxygenated groups, leading to a more functionalized and reactive material.

The XRD pattern of the MP shows the typical strong crystalline reflections near ~21.5° and ~24°, indicating well-ordered orthorhombic PE domains [24] (Figure 1e). When cobalt is immobilized on the MP, these main PE peaks remain, but the Co–MP trace develops a subtle shoulder at ~19.3°, suggesting slight disruption of PE chain packing or the presence of a very weak, poorly crystalline cobalt-related contribution. Additional broad intensity at higher angles is consistent with dispersed, low-crystallinity cobalt species rather than well-defined cobalt oxide phases. Overall, the Co–MP pattern reflects preserved PE crystallinity with minor structural perturbation and the formation of poorly ordered cobalt-containing domains.

BET surface area analysis showed very low specific surface areas for MP (0.69 m^2^·g^−1^) and Co–MP (0.56 m^2^·g^−1^), which is fully consistent with the inherently nonporous nature of polyethylene-based MPs. The slight decrease after cobalt immobilization reflects partial surface coverage by cobalt species, resulting in a minor reduction of nitrogen-accessible sites without inducing any meaningful pore blockage. This is supported by the fact that the BET instrument used can reliably measure surface areas as low as 0.01 m^2^·g^−1^, while the observed difference (0.13 m^2^·g^−1^) lies within the typical measurement uncertainty for low-surface-area materials (±0.15–0.20 m^2^·g^−1^). Thus, the variation is not considered significant. The results indicate that cobalt was deposited as a thin, well-dispersed layer rather than forming bulk agglomerates, preserving the overall surface accessibility of the MP substrate. Importantly, catalytic activity in this system is governed not by the polymer’s intrinsic surface area but by the accessibility and redox functionality of the surface-bound cobalt species, which act as the active centers for PMS activation and nitrate reduction.

In addition to the surface-area changes, the pore structural analysis provides further evidence of Co-MP interactions. The pristine MP exhibited a mean pore diameter of 12.23 nm, whereas the Co–MP sample displayed an increased value of 14.8 nm. This upward shift in average pore size, occurring alongside a decrease in BET surface area from 0.69 m^2^ g^−1^ to 0.56 m^2^ g^−1^, is characteristic of selective pore blockage rather than simple external surface coverage. As shown in the Barrett–Joyner–Halenda (BJH) pore-size distribution curves (Figure 1f), the overall shapes of the MP and Co–MP distributions remain broadly similar and partially overlapping; however, the Co–MP curve exhibits a reduced pore volume and a subtle shift toward larger mesopores. These combined changes indicate that cobalt deposition preferentially obstructs smaller mesopores within the polymer matrix, effectively removing them from the detectable distribution and thereby increasing the average pore diameter. Although polyethylene is generally considered nonporous, the BJH results for this MP material confirm the presence of a measurable mesoporous network. Thus, the observed BET–BJH trends support a mechanism of partial internal pore filling rather than mere external cobalt coating or structural collapse, while larger pores remain open to sustain mass transfer and catalytic activity.

While SEM, FTIR, EDX, and XRD collectively confirm successful cobalt deposition and surface modification of the microplastics, these techniques primarily provide morphological and elemental information rather than direct evidence of chemical bonding or electronic structure. The observed shifts and intensity changes in the FTIR spectra imply interactions between cobalt species and oxygen-containing groups (e.g., –OH, –C=O) on the MP surface, but such evidence remains indirect. To unambiguously determine the oxidation states and local chemical environment of cobalt, X-ray Photoelectron Spectroscopy (XPS) would be required. XPS could distinguish between Co^2+^ and Co^3+^ states, quantify surface oxygen species (O^2−^, O–H, adsorbed O*, defect oxygen), and detect binding-energy shifts in the O 1s and C 1s regions indicative of Co–O or Co–C coordination structures. This surface-level electronic information is essential for understanding PMS activation behavior and would greatly strengthen the mechanistic interpretation of cobalt-mediated nitrate reduction. Although XPS was not available for the present study, it remains a priority for future work. For additional discussion on how oxygen species and transition-metal electronic structure influence PMS activation pathways, readers may refer to Sun et al. (2023), who reported a detailed analysis of NiOOH–CuO nano-heterostructures [25].

### 2.2. Control Experiments and Preliminary Evaluation

**Figure 2:**
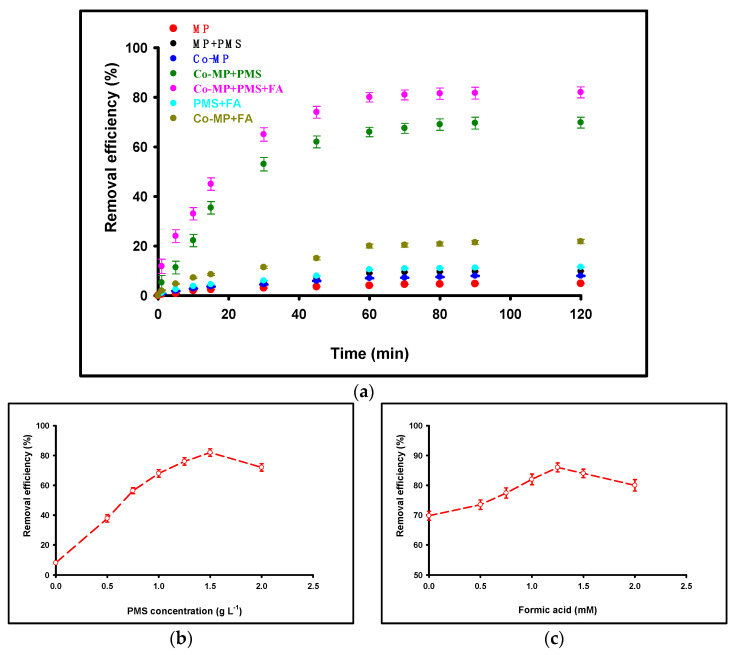
Nitrate removal performance under various control and operational conditions. (**a**) Comparison of control systems: MP, M + PMS, PMS + FA, Co–MP, Co–MP + FA, Co–MP + PMS, and the complete Co–MP + PMS + FA system. (**b**) Effect of PMS concentration on nitrate degradation. (**c**) Effect of FA concentration on nitrate degradation. All experiments were conducted under the following conditions: initial [NO_3_^−^] = 50 mg L^−1^, pH 6, reaction time 120 min, temperature 25 ± 2 °C, Co(II) = 60 mg L^−1^, and MP dosage = 1.0 g L^−1^.

To elucidate the contribution of individual components to nitrate degradation, a series of control experiments were conducted under identical conditions (initial [NO_3_^−^] = 50 mg L^−1^, pH = 6, reaction time = 120 min, and temperature = 25 ± 2 °C). Each experiment employed a fixed cobalt loading of 60 mg L^−1^ (as Co^2+^ equivalent) and a Co–MP dosage of 1.0 g L^−1^, unless otherwise specified. The measured nitrate removals were: MP = 4.8%; MP + PMS = 9.8%; PMS + FA = 11.5%; Co–MP = 7.95%; Co–MP + FA = 21.8%; Co–MP + PMS = 69.8%; and Co–MP + PMS + FA = 82.0%. As shown in Figure 2a, negligible nitrate removal (<5%) occurred in the absence of PMS, indicating that adsorption on the MP surface contributed minimally to nitrate transformation. When PMS (1.5 g L^−1^) was added without cobalt or formic acid, only a slight decrease in nitrate concentration (<10%) was observed, attributable to the weak oxidative potential of PMS alone. The PMS + FA control produced only a modest improvement relative to MP + PMS, indicating that formic acid alone can interact with PMS to generate limited reactive species but cannot drive efficient, multi-electron nitrate reduction under these conditions. Similarly, the use of Co–MP without PMS resulted in limited nitrate reduction (~8%), reflecting the low catalytic activity of cobalt sites in the absence of an oxidant. Co–MP + FA led to a larger, but still partial, nitrate removal (≈21.8%), consistent with formic acid donating electrons to surface cobalt sites but with inefficient catalyst regeneration in the absence of PMS. In contrast, the combined Co–MP + PMS system exhibited a substantial increase in nitrate removal (~70%), confirming that cobalt-mediated PMS activation was the predominant degradation pathway. The further introduction of formic acid (1 mM) as a sacrificial electron donor enhanced nitrate removal to nearly 82%, highlighting its role in accelerating the Co^2+^/Co^3+^ redox cycle and sustaining continuous radical generation. These results clearly differentiate the relative contributions of adsorption, catalytic oxidation, and redox-mediated degradation. The minimal removal observed in the absence of PMS or Co–MP confirms that nitrate elimination in this system proceeds mainly through radical-driven oxidative pathways rather than surface adsorption. The enhancement achieved with formic acid addition underscores its synergistic role in promoting PMS activation and maintaining high catalytic turnover efficiency. To determine the optimum concentration of PMS for nitrate degradation, a series of batch experiments were conducted using different PMS dosages (0.5, 0.75, 1.0, 1.25, 1.5, 1.75, and 2.0 g·L^−1^) under identical before conditions. As illustrated in Figure 2b, nitrate removal efficiency increased progressively with PMS concentration from 0 to 1.5 g·L^−1^, improving from 8% to 82%. However, further increasing PMS beyond 1.5 g·L^−1^ resulted in a decline in efficiency to 73.4%. This observation suggests that 1.5 g·L^−1^ is the optimum PMS concentration for nitrate removal, as excessive PMS may lead to radical self-quenching or parasitic reactions (e.g., ${SO_{4}^{\cdot }}^{-}$ and ^•^OH recombination), thereby reducing the effective oxidizing capacity of the system. To identify the optimal concentration of formic acid (FA) for nitrate degradation, a series of batch experiments were performed using different FA dosages of 0.5, 0.75, 1.0, 1.25, 1.5, and 2.0 mM under identical initial conditions. As illustrated in Figure 2c, nitrate removal efficiency increased progressively with formic acid (FA) concentration from 0 to 1.25 mM, rising from 69.8% to 86% due to the enhanced regeneration of Co^2+^ and sustained radical production. However, further increasing the FA concentration beyond 1.25 mM (up to 2 mM) led to a decline in efficiency from 86% to 80%, which can be attributed to radical scavenging and system quenching effects.

### 2.3. Nitrate Degradation Performance of the Co–MP/PMS System

**Figure 3:**
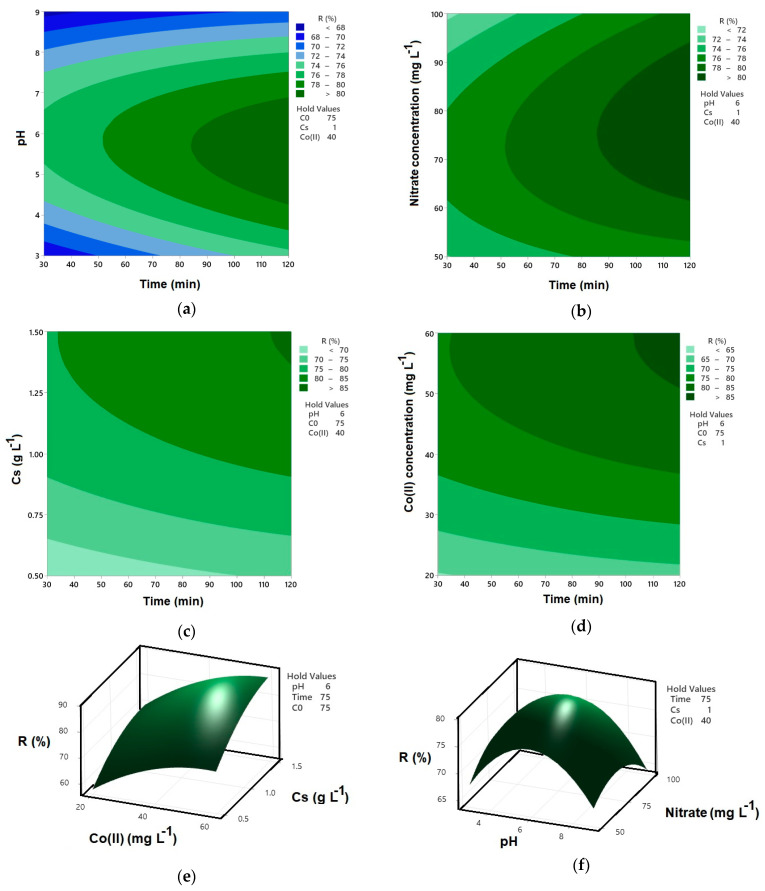
Contour and surface plots for Co(II)-MP/PMS/FA system: (**a**) interaction of reaction time and pH on R; (**b**) interaction of reaction time and initial nitrate concentration on R; (**c**) interaction of reaction time and catalyst dosage on R; (**d**) interaction of reaction time and Co(II) concentration on R; (**e**) interaction of catalyst dosage and Co(II) concentration on R; and (**f**) interaction of pH and initial nitrate concentration on R.

Figure 3a illustrates the combined effect of pH and reaction time on nitrate removal efficiency (R%) in the Co–MP/PMS/FA system at fixed conditions (initial nitrate concentration = 75 mg L^−1^, catalyst dosage = 1.0 g L^−1^, and Co(II) = 40 mg L^−1^). As shown, nitrate removal increases with both longer reaction time and near-neutral pH. The highest efficiency (>80%) occurs around pH 6 and 100–120 min, indicating optimal PMS activation and sustained radical generation. At lower pH (<4), excessive proton concentration suppresses sulfate radical formation [26], whereas under alkaline conditions (>8), cobalt deactivation and PMS decomposition limit oxidation capacity [27]. The nearly elliptical contour centered at pH 6 and 100 min highlights the strong interaction between these factors, confirming that near-neutral pH and sufficient contact time are critical for maximum nitrate degradation in the Co–MP/PMS/FA system.

Figure 3b illustrates the combined effect of initial nitrate concentration and reaction time on nitrate removal efficiency (R%) in the Co–MP/PMS/FA system at fixed conditions (pH = 6, catalyst dosage = 1.0 g·L^−1^, and Co(II) = 40 mg·L^−1^). Nitrate removal efficiency increases with longer reaction time but decreases with higher initial nitrate concentration. At lower nitrate concentrations (50–70 mg·L^−1^), removal exceeds 80% after 100–120 min, reflecting sufficient oxidant availability and sustained radical generation. In contrast, higher initial concentrations (>90 mg·L^−1^) result in lower efficiency (<72%) due to competition for reactive radicals and a reduced PMS-to-pollutant ratio [28]. The contour pattern indicates that optimal nitrate degradation occurs at moderate initial concentrations (≈70–80 mg·L^−1^) and longer reaction times (>90 min), confirming that pollutant load significantly influences the overall oxidation performance of the Co–MP/PMS/FA system.

Figure 3c illustrates the synergistic relationship between catalyst dosage and reaction time for nitrate degradation under the specified conditions: pH 6, an initial nitrate concentration of 75 mg·L^−1^, and a Co(II) concentration of 40 mg·L^−1^. The plot demonstrates that increasing the catalyst dosage significantly reduces the time required to achieve high nitrate removal efficiency. This is attributed to a greater availability of active sites on the Co–MP catalyst, which enhances the activation of PMS and promotes more rapid generation of sulfate radicals [29]. The contours show that at a lower catalyst dosage, a longer reaction time is necessary to reach a target removal level, whereas a higher dosage accomplishes the same result in a considerably shorter duration. This inverse relationship underscores that sufficient catalyst loading is crucial for optimizing the process kinetics, ensuring efficient PMS activation and sustained radical-driven oxidation under continuous stirring.

Figure 3d reveals a direct relationship between Co(II) concentration and nitrate degradation rate, where higher Co(II) levels lead to a significantly faster achievement of high removal efficiency under fixed conditions (1 g L^−1^ catalyst, pH 6, 75 mg L^−1^ NO_3_^−^). Specifically, at lower Co(II) concentrations, reaching a removal efficiency exceeding 85% requires a substantially longer reaction time. Conversely, as the Co(II) concentration increases, the same high level of degradation is achieved in a much shorter duration. This is because the Co(II) ions in solution work synergistically with the cobalt immobilized on the MP, providing additional active sites for the activation of PMS. This enhanced activation accelerates the generation of sulfate radicals, thereby speeding up the oxidation of nitrate [21]. The contour lines clearly demonstrate that increasing the Co(II) concentration is a highly effective strategy for intensifying the reaction kinetics and reducing the necessary treatment time.

As shown in Figure 3e, a strong synergistic effect between Co(II) concentration and catalyst dosage (Cs) governs the nitrate removal efficiency at a fixed pH of 6, an initial nitrate concentration of 75 mg L^−1^, and a reaction time of 75 min. The plot shows that nitrate removal increases with both higher Co(II) concentration and greater catalyst dosage. However, the highest efficiencies are achieved not by maximizing just one parameter, but by a balanced combination of both. At low catalyst dosages, even high Co(II) concentrations yield only moderate removal, as the limited solid catalyst surface area restricts the number of available activation sites. Conversely, a high catalyst dosage with low Co(II) concentration is also sub-optimal, suggesting that the immobilized cobalt alone is insufficient for maximum PMS activation within the given time [5].

Figure 3f illustrates the influence of pH and initial nitrate concentration on nitrate removal efficiency (R%) under fixed conditions of 40 mg L^−1^ Co(II), 1 g L^−1^ catalyst dosage, and a 75 min reaction time. The surface plot clearly shows that the highest removal efficiencies (R% > 75%) are consistently achieved within a narrow, near-neutral pH window, centered around pH 6 to 7, across all tested initial nitrate concentrations. This pH range is optimal for the Co(II)/Co(III) redox cycling, facilitating efficient PMS activation and sustained radical generation [30]. Furthermore, the efficiency is inversely related to the initial nitrate concentration. At a fixed pH, the removal percentage decreases as the initial nitrate load increases from 50 mg L^−1^ to 100 mg L^−1^. For instance, at the optimal pH of ~6.5, the removal percentage drops from over 75% at 50 mg L^−1^ nitrate to approximately 65–67% at 100 mg L^−1^ nitrate. This decline occurs because a higher pollutant load consumes the finite quantity of reactive radicals generated, effectively diluting the oxidative capacity of the system.

### 2.4. Process Optimization and Statistical Analysis of Nitrate Degradation

Table 1 shows the Analysis of Variance (ANOVA) results, revealing the relative influence of each operational parameter on nitrate removal efficiency. Catalyst dosage (Cs) and Co(II) concentration emerge as the dominant and highly significant factors, with high F-values of 53.81 and 70.72, respectively, and *p*-values of 0.000. This indicates that the variance they explain is overwhelmingly large compared to the experimental noise, providing definitive evidence that they are the primary drivers of the degradation process. Because cobalt immobilization varies with solution chemistry (including initial Co(II) concentration and pH), the actual cobalt loading on MP is not constant across RSM conditions. For this reason, MP dosage and added dissolved Co(II) concentration were treated as independent variables: MP dosage determines the amount of heterogeneous polymeric support available, whereas dissolved Co^2+^ governs cobalt availability for immobilization and for the homogeneous Co(II)/Co(III) redox activation of PMS.

Reaction time was also a statistically significant factor (*p* = 0.021), confirming that longer contact times enhance removal efficiency, although its influence was substantially weaker than that of the catalyst dosage and Co(II) concentration. In contrast, the linear effects of pH and initial nitrate concentration (C_0_) are not statistically significant, with high *p*-values of 0.156 and 0.861, respectively. This means that, within the ranges tested, their individual impact on the response cannot be distinguished from random variation. Furthermore, the highly significant quadratic term for pH reveals a critical curvilinear relationship, indicating that removal efficiency reaches an optimum value at a specific pH level rather than changing linearly. Conversely, all two-way interaction effects between the factors were found to be statistically insignificant, suggesting that the effect of one factor does not depend on the level of another in this experimental domain. Based on the ANOVA for the experiment, the statistical model for nitrate degradation is highly significant, as evidenced by the very low *p*-value for the overall model (0.000). This indicates that the model effectively explains the relationship between the operational parameters and the nitrate removal efficiency.

The statistical analysis revealing that the linear effects of pH and initial nitrate concentration (C_0_) are not significant within the tested model holds considerable positive implications for the practical deployment of this Co(II)-MP/PMS/FA system. The non-significant effect of pH, particularly within the studied range, is a highly desirable trait for a water treatment technology. Many advanced oxidation processes, especially those reliant on hydroxyl radicals, are severely constrained by a narrow optimal pH range, often requiring costly and continuous pH adjustment in real-world applications [31]. The insensitivity of this system to pH variation suggests a robust operational window. This robustness can be attributed to the nature of the sulfate radical, which is active over a broader pH range than the hydroxyl radical, and the role of formic acid in maintaining the catalytic cycle. Furthermore, the lack of a statistically significant linear effect for the initial nitrate concentration indicates that the process possesses a substantial oxidative capacity. The system can degrade a given percentage of nitrate with similar efficiency across a range of initial concentrations (50–100 mg L^−1^). This suggests that for a fixed reaction time, the system can handle variable pollutant loads, a common scenario in real wastewater or contaminated groundwater.

The regression equation derived from the second-order polynomial model describes the relationship between nitrate removal efficiency (*R*, %) and the significant operational parameters in un-coded units. The fitted empirical model can be expressed as follows:

(1) $$R\;\left(\%\right)=\;-60.3+0.028\times t+45.8\times C_{S}+1.17\times Co\left(II\right)-0.968\times \left(pH\times pH\right)$$

where t is the reaction time (min), $C_{S}$ is the catalyst dosage (g·L^−1^), and Co(II) is the cobalt concentration (mg·L^−1^). This model indicates that nitrate removal efficiency increases with longer reaction time, higher catalyst loading, and greater cobalt concentration, while excessively high or low pH values negatively influence the process efficiency due to the quadratic dependence on pH. The positive coefficients for t, $C_{S}$, and Co(II) confirm their synergistic roles in promoting PMS activation and radical generation, whereas the negative quadratic pH term reflects the narrow optimal range around near-neutral conditions where cobalt species remain catalytically active and PMS decomposition is most efficient. The model exhibited a strong correlation between experimental and predicted data, with a coefficient of determination (R^2^) of 96.31% and an adjusted R^2^ of 90.15%, indicating excellent predictive accuracy and reliability. These results confirm that the selected parameters significantly influence nitrate degradation performance in the Co–MP/PMS/FA system, and the quadratic model provides a robust representation of the experimental response behavior.

Although the contour plots (Figure 3a,e) display curved surfaces that may seem to indicate interaction effects, the ANOVA results show that none of the two-factor interaction terms were statistically significant (*p* > 0.05). The observed curvature originates from the significant quadratic effect of pH^2^, which introduces non-linear response behavior even in the absence of significant interactions. Only reaction time, catalyst dosage, and Co(II) concentration were significant linear factors, whereas the quadratic effect of pH was the only higher-order term affecting the response. As a result, the contour plots show curvature driven solely by the pH^2^ contribution rather than by true interaction effects. Therefore, the graphical representation is consistent with the statistical analysis and does not indicate a model-fitting conflict.

**Figure 4:**
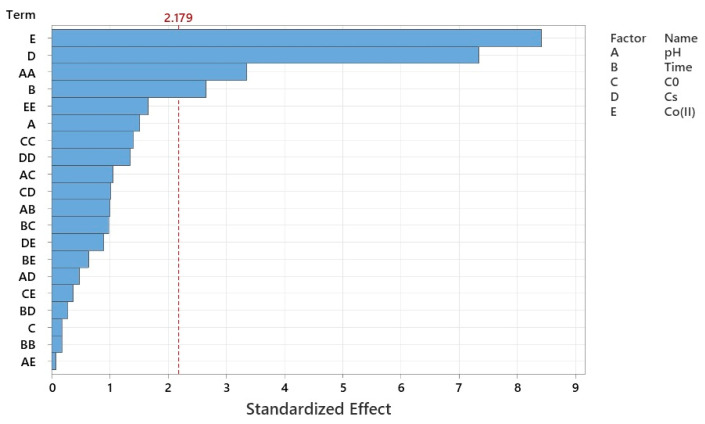
Pareto chart of standardized effects.

The Pareto chart of standardized effects (Figure 4) illustrates the relative significance of each operational parameter on nitrate removal efficiency within the Co–MP/PMS/FA system. The vertical red line at the critical value (t = 2.179) represents the 95% confidence threshold; any factor whose bar extends beyond this line is statistically significant. As shown, Co(II) concentration (E) exerts the most pronounced influence on nitrate removal, followed closely by catalyst dosage (D). Both parameters significantly affect PMS activation efficiency, as higher cobalt loading and catalyst mass enhance the generation of reactive radicals (${SO_{4}^{\cdot }}^{-}$ and ^•^OH) and provide more active surface sites. Reaction time (B) also shows a notable positive effect, confirming that extended contact allows for more complete nitrate transformation. Among the quadratic and interaction terms, the pH^2^ (AA) effect is significant, indicating that the process is highly sensitive to pH variation, with efficiency declining at both acidic and alkaline extremes. The linear term of pH (A) also crosses the threshold, emphasizing that near-neutral pH conditions are optimal for maintaining cobalt stability and effective PMS activation. In contrast, initial nitrate concentration (C) and higher-order interactions such as AE, BD, and BE exhibit minimal effects, suggesting that within the tested range, substrate concentration plays a lesser role compared to catalyst-related parameters. Overall, the Pareto analysis confirms that Co(II) concentration and catalyst dosage are the dominant factors, followed by reaction time and pH, while initial nitrate concentration has a relatively smaller impact on nitrate degradation efficiency. This hierarchy aligns well with the regression model, validating that catalytic and redox activation parameters primarily govern the Co–MP/PMS/FA performance.

The model predicts a maximum nitrate removal of R = 90.93% (predicted) at the following operating point: pH = 5.5, reaction time = 120 min, initial nitrate concentration = 73.7 mg·L^−1^, catalyst dosage C_s_ = 1.50 g·L^−1^, and cobalt loading Co(II) = 60 mg·L^−1^. The desirability function value associated with this solution is d = 0.8423, indicating a high overall agreement with the optimization objective (maximize R while respecting the experimental bounds). These settings lie within the experimental domain used to fit the quadratic model and are consistent with the mechanistic expectation that moderate acidity (near-neutral), long contact time, high catalyst/Co loading and moderate pollutant load favor PMS activation and radical-driven nitrate transformation. To evaluate the accuracy of the CCD–RSM model in predicting the optimal operational conditions for nitrate removal, batch validation experiments were conducted under the model-predicted settings (pH ≈ 5.5, reaction time = 120 min, initial nitrate concentration C_0_ ≈ 73.7 mg·L^−1^, catalyst dosage C_s_ = 1.5 g·L^−1^, and Co(II) concentration = 60 mg·L^−1^). All tests were performed in triplicate to ensure reproducibility. The experimental nitrate removal efficiencies were 91.4%, 89.8%, and 90.6%, yielding an average removal efficiency of 90.6 ± 0.8% (RSD = 0.9%). These results are in excellent agreement with the model-predicted value of 90.93%, confirming the reliability and predictive capability of the CCD–RSM design. The close correlation between predicted and experimental values validates the adequacy of the quadratic regression model and demonstrates that the selected parameters effectively describe the nitrate degradation behavior in the Co–MP/PMS system.

### 2.5. Nitrogen Species Analysis and Nitrogen Mass Balance

**Figure 5:**
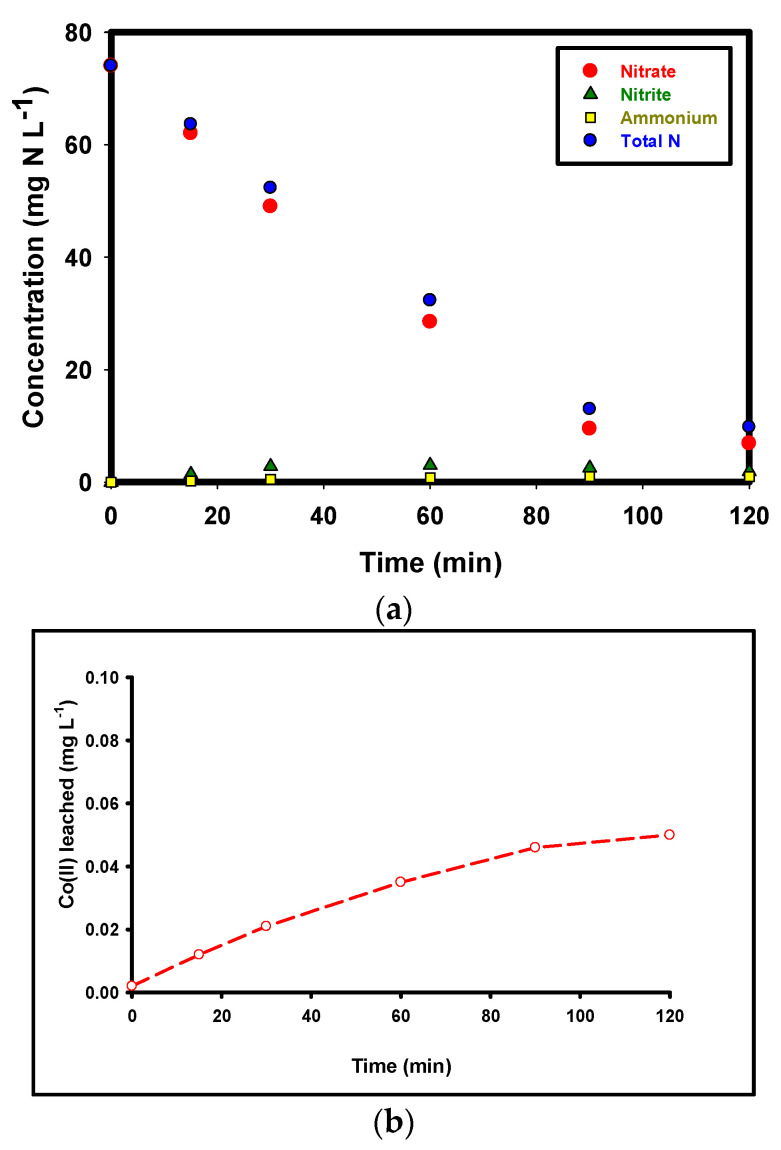
(**a**) Temporal variations in nitrate-N, nitrite-N, ammonium-N, and total nitrogen (TN) during the Co–MP/PMS/FA process. Experimental conditions: pH = 5.5, reaction time = 120 min, C_0_ = 74 mg·L^−1^, C_s_ = 1.5 g·L^−1^, Co(II) = 60 mg·L^−1^, PMS = 1.5 g·L^−1^, FA = 1 mM; and (**b**) Co(II) leaching in solution.

The changes in nitrogen species concentrations (nitrate, nitrite, ammonium, and total nitrogen) as nitrogen mass concentrations (mg N L^−1^) in the effluent during the Co–MP/PMS/FA process over 0–120 min, under optimal operating conditions (pH = 5.5, reaction time = 120 min, C_0_ = 74 mg·L^−1^, C_s_ = 1.5 g·L^−1^, Co(II) = 60 mg·L^−1^, PMS = 1.5 g·L^−1^, FA = 1 mM) are shown in Figure 5a. Since Total Nitrogen (TN) analysis quantifies only dissolved nitrogen species, the observed reduction indicates that a large portion of nitrogen was removed from the aqueous phase but does not directly confirm the formation of gaseous nitrogen products. To further interpret these results, a quantitative nitrogen mass balance was calculated by summing the measured concentrations of NO_3_^−^, NO_2_^−^, NH_4_^+^, and TN at the beginning and end of the reaction. The nitrate concentration exhibits a rapid and continuous decline throughout the reaction, decreasing from 74 mg·L^−1^ at 0 min to 6.9 mg·L^−1^ by 120 min, corresponding to a 90.6% removal of the initial nitrate load. This sharp decrease demonstrates the high catalytic efficiency and sustained activity of the Co–MP/PMS/FA system for nitrate degradation. Nitrite shows a transient accumulation, increasing from 0.0 mg·L^−1^ to a maximum of 3.0 mg·L^−1^ at 60 min before gradually declining to 1.9 mg·L^−1^ by 120 min. This trend confirms that nitrite acts as a key intermediate during the stepwise reduction in nitrate, being first formed and subsequently transformed into other nitrogenous products. Ammonium generation was also detected but remained at low levels, rising slowly from 0.0 to 1.0 mg·L^−1^ by 90 min and then stabilizing, suggesting that ammonium formation is a minor or short-lived pathway and does not accumulate significantly in the solution. Total nitrogen (TN) concentration decreased markedly from an initial 74 mg·L^−1^ to 9.8 mg·L^−1^ after 120 min, representing an 86.7% overall reduction. This substantial decline in TN confirms that nitrogen removal does not occur solely through transformation into other dissolved forms (such as NO_2_^−^ or NH_4_^+^) but rather through conversion to gaseous nitrogen species [32,33]. The concurrent decreases in nitrate and total nitrogen, combined with the transient presence of nitrite, strongly indicate that the Co–MP/PMS/FA system promotes an effective denitrification process, in which nitrate is ultimately reduced to gaseous products such as N_2_ or N_2_O that escape from the aqueous phase.

The difference between the initial and final TN values represents a missing nitrogen fraction, which strongly suggests volatilization as N_2_ or N_2_O; however, this interpretation remains indirect. Although a substantial decrease in total nitrogen (86.7%) was observed, TN measurements quantify only dissolved nitrogen species and do not directly measure gaseous products. The combined observations of high TN removal, transient nitrite accumulation, and minimal ammonium formation are consistent with a denitrification pathway, but alternative routes—such as the formation of trace organic nitrogen or other dissolved intermediates below detection limits—cannot be fully excluded. Because headspace gases were not collected or analyzed, the formation of N_2_ or N_2_O cannot be conclusively confirmed in the present study. A fully closed nitrogen mass balance, including direct detection of gaseous species, is therefore required for unambiguous verification. Future work will incorporate headspace sampling and gas chromatography (e.g., GC-TCD or GC-MS) to directly quantify N_2_ and N_2_O and conclusively resolve the nitrogen transformation pathway.

The measured dissolved Co(II) concentrations show a clear, time-dependent release from the Co–MP catalyst: very low at t = 0 (≈0.002 mg·L^−1^), rising during the reaction and approaching a near steady value of ~0.048–0.05 mg·L^−1^ by 120 min. This profile is typical for heterogeneous, surface-anchored metal catalysts operating under oxidizing conditions. An initial rapid release reflects the desorption or oxidative dissolution of weakly bound, labile Co species on the outermost surface; the subsequent slower rise toward a plateau suggests that the remaining cobalt is more strongly bound and less susceptible to further oxidative leaching during a single run. Mechanistically, Co leaching under PMS activation is promoted by (i) oxidative conversion of surface Co(II) to higher oxidation states (Co^3+^/Co^4+^) that are more soluble, (ii) local acidic microenvironments generated during radical reactions, and (iii) direct chemical attack by strong oxidants or radical species. The observed magnitudes (~0.05 mg·L^−1^ after 120 min) are small compared with the initial loading (≈60 mg·L^−1^), indicating that the vast majority of Co remains on the support; therefore, catalysis is predominantly heterogeneous. From an environmental and regulatory perspective, the reported leaching level is low but non-negligible. To mitigate Co(II) release, we recommend enhancing Co anchoring via surface functionalization (e.g., PDA coating or carboxylation) or forming firmly attached oxide phases, operating at near-neutral pH (≈6–6.5), and using split PMS dosing to reduce instantaneous oxidative stress.

In addition to catalytic performance, the practical recovery of the Co–MP material is essential for ensuring the environmental validity of the dual-remediation concept. Because the microplastic support is based on low-density polyethylene (ρ ≈ 0.95 g cm^−3^), the composite remains buoyant during treatment, enabling straightforward flotation-based recovery through decantation or surface skimming. This inherent buoyancy minimizes the risk of secondary microplastic release. Future development of the material may also incorporate magnetically responsive components (e.g., Fe_3_O_4_), allowing magnetic separation as an efficient and scalable retrieval method. Recovered Co–MP particles can be rinsed and reused, whereas spent materials can be consolidated for controlled disposal or recycling. Integrating these recovery strategies is essential for the environmental practicality of the process.

### 2.6. Chemical Pathway for Nitrate Degradation

The concentration profiles of nitrate, nitrite, ammonium, and total nitrogen (Section 2.5) indicate that the Co–MP/PMS/FA system promotes a stepwise reductive denitrification process rather than direct oxidation by sulfate or hydroxyl radicals. The transient formation of nitrite, minimal accumulation of ammonium, and overall 86.7% reduction in total nitrogen clearly suggest that nitrate (NO_3_^−^) is sequentially converted into gaseous nitrogen species (NO, N_2_O, N_2_). This observation is inconsistent with a purely radical-oxidation pathway, since both sulfate (SO_4_^•−^, E° = +2.6 V) and hydroxyl (^•^OH, E° = +2.8 V) radicals are strong oxidants [5,34,35], thermodynamically incapable of reducing nitrate. Instead, the experimental results support a coupled oxidative–reductive mechanism in which formic acid acts as an electron donor [36,37] and the cobalt redox cycle mediates electron transfer to nitrate. In this mechanism, formic acid (HCOOH) is oxidized to CO_2_ while donating electrons to the cobalt species immobilized on the microplastic surface. The cobalt catalyst shuttles these electrons to nitrate via a sequence of redox transformations:

HCOOH → CO_2_ + 2H^+^ + 2e^−^ (2)

Co(III) + e^−^ → Co(II) (3)

NO_3_^−^ + 2e^−^ + 2H^+^ → NO_2_^−^ + H_2_O (4)

2NO_2_^−^ + 4H^+^ + 2e^−^ → 2NO + 2H_2_O (5)

2NO + 2e^−^ + 2H^+^ → N_2_O + H_2_O (6)

N_2_O + 2H^+^ + 2e^−^ → N_2_ + H_2_O (7)

Overall, electrons derived from formic acid flow through the Co(II)/Co(III) couple to nitrate intermediates, producing molecular nitrogen as the terminal product. The function of peroxymonosulfate (PMS, HSO_5_^−^) in this system is not to generate oxidative radicals that attack nitrate, but to re-oxidize reduced cobalt species (e.g., Co(I) or surface Co–H) back to catalytically active Co(II)/Co(III) states, thus maintaining the electron-transfer cycle:

Co(I) + HSO_5_^−^ → Co(II) + SO_4_^2−^ + OH^−^ (8)

Co–H + HSO_5_^−^ → Co(II) + SO_4_^2−^ + H_2_O (9)

**Figure 6:**
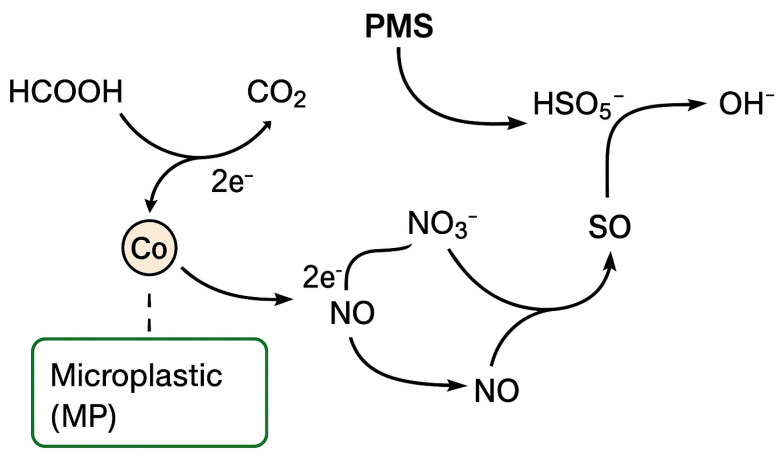
Proposed coupled oxidative–reductive mechanism for nitrate transformation in the Co–MP/PMS/FA system.

Through this PMS-assisted regeneration, cobalt species continuously mediate the flow of electrons from formic acid to nitrate without substantial cobalt leaching. This integrated redox cycle explains the high nitrate and total nitrogen removal efficiencies observed under near-neutral pH conditions, where both cobalt stability and PMS activation are optimal. Similar coupled oxidative–reductive systems involving cobalt- or other transition-metal-mediated pollutant transformation in the presence of formic acid or persulfate have been reported in recent studies [28,38,39]. A schematic reaction diagram for nitrate degradation is presented in Figure 6.

While the nitrogen balance strongly suggests conversion to gaseous nitrogen species, a complete mass balance including headspace measurements is needed to definitively confirm the identity and distribution of the gaseous products. Future studies will incorporate GC-based analysis to close the nitrogen mass balance. The specific oxygen species associated with the Co–MP surface—such as lattice oxygen, surface hydroxyl oxygen, and chemisorbed oxygen—likely play an important role in PMS activation, as reported in recent PMS-driven redox-catalyst systems. These oxygen functionalities can modulate electron transfer between Co(II)/Co(III) and PMS, influencing the formation of reactive intermediates and enabling nitrate reduction. Comprehensive XPS analysis, similar to that employed in NiOOH-CuO heterostructure studies [25], is needed in future work to determine these oxygen environments and further validate the proposed redox mechanism.

## 3. Materials and Methods

### 3.1. Materials

All chemicals used in this study were of analytical grade and used without further purification. Potassium nitrate (KNO_3_) was employed as the model nitrate pollutant. Cobalt(II) chloride hexahydrate (CoCl_2_·6H_2_O), Peroxymonosulfate (PMS, 2KHSO_5_·KHSO_4_·K_2_SO_4_,), ethanol, and formic acid were obtained from Merck (Darmstadt, Germany). High-purity deionized (DI) water was used in all solution preparations. Commercial polyethylene (PE) MPs, with an average particle size of 100–500 µm and density of approximately 0.95 g cm^−3^, were used as the support material. Prior to use, the MPs were thoroughly washed with ethanol and DI water, ultrasonicated for 30 min to remove surface impurities, and dried at 60 °C for 12 h.

### 3.2. Preparation of Co-Immobilized Microplastics (Co–MP Catalyst)

Cobalt was immobilized onto the surface of PE-MPs through an adsorption–deposition method. In a typical procedure, 0.5–1.5 g of pre-treated MPs were dispersed in 200 mL aqueous solutions containing 20–60 mg L^−1^ of Co(II) (prepared from CoCl_2_·6H_2_O) under continuous magnetic stirring at 300 rpm for 6 h at room temperature. The suspension was then left to equilibrate for an additional 12 h to enhance cobalt adsorption and surface interaction with the polymer matrix. The cobalt immobilization step was performed at pH 6.0, which was adjusted and maintained throughout the adsorption period. This pH was selected to ensure favorable electrostatic interaction and coordination between Co^2+^ ions and functional groups on the MP surface while preventing cobalt precipitation. The cobalt loading reported for the characterized Co–MP material (5.2 wt%) corresponds specifically to the condition where the initial Co(II) concentration in solution was 60 mg L^−1^. Because cobalt immobilization efficiency depends on solution chemistry (e.g., Co(II) concentration and pH), cobalt loading varies under different RSM conditions. Therefore, in optimization experiments, “ catalyst dosage” refers strictly to the mass of MP introduced (g L^−1^), rather than to a fixed cobalt mass. After the adsorption step, the cobalt-loaded MPs were separated by filtration, rinsed repeatedly with DI water to remove unbound cobalt ions, and dried at 60 °C for 12 h. The dried material was then subjected to mild thermal treatment at 120 °C for 2 h to improve cobalt adhesion and structural stability on the MP surface. The thermal treatment at 120 °C was selected to facilitate dehydration of the MP–Co complex and promote coordination between Co^2+^ species and surface oxygen functionalities without exceeding the melting point of polyethylene (~130–140 °C). This temperature is below the threshold at which polyethylene undergoes morphological deformation, ensuring that the MP matrix remains intact while enabling effective cobalt anchoring.

### 3.3. Characterization of the Catalyst

The physicochemical properties of the prepared Co–MP catalysts were comprehensively characterized to elucidate their structural, morphological, and surface characteristics. FTIR was performed using a Bruker Tensor 27 spectrometer (Bruker Optics GmbH, Ettlingen, Germany) in the range of 400–4000 cm^−1^ to identify functional groups and confirm the interaction between cobalt species and the MP surface. Surface morphology and elemental composition were analyzed using SEM (TESCAN VEGA3) (TESCAN, Brno, Czech Republic) coupled with EDAX. The crystalline phase structures of the samples were examined using XRD with a Philips X’PERT MPD diffractometer (PANalytical, EA Almelo, The Netherlands). Specific surface area, pore volume, and pore diameter were determined by nitrogen adsorption–desorption isotherms using the BET method with a Belsorp mini II instrument (MicrotracBEL, Osaka, Japan). Cobalt loading on the MP surface was quantified by Inductively Coupled Plasma–Optical Emission Spectroscopy (ICP-OES; PerkinElmer Optima 7000 DV, PerkinElmer, Inc., Shelton, CT, USA) after acid digestion of a known mass of catalyst in 10 mL of 5% HNO_3_.

### 3.4. Experimental Procedure

Two experimental frameworks were used. (i) Mechanistic characterization and control experiments were carried out under one fixed condition (initial [NO_3_^−^] = 50 mg L^−1^, pH 6, reaction time 120 min, temperature 25 ± 2 °C), with 60 mg L^−1^ Co(II) and 1.0 g L^−1^ MP. The cobalt loading (5.2 wt%) reported in Section 2.1 corresponds exclusively to this condition. (ii) RSM optimization experiments varied MP dosage (g L^−1^) and added dissolved Co(II) concentration (mg L^−1^) as independent variables. Because cobalt immobilization is not constant across RSM conditions, MP dosage is used as the catalyst mass parameter, while Co(II) concentration refers only to dissolved cobalt initially present in solution. Batch experiments were conducted to evaluate nitrate transformation in the Co–MP/PMS system. Each experiment was performed in a 250 mL Erlenmeyer flask containing 100 mL of nitrate solution (50–100 mg L^−1^). The required amounts of Co–MP catalyst (0.5–1.5 g L^−1^, containing 20–60 mg L^−1^ of Co(II)) and PMS (0.5–2.0 g L^−1^) were added under continuous magnetic stirring at 300 rpm to ensure homogeneous dispersion. To enhance PMS activation, a small quantity of formic acid (HCOOH; 0.5–2 mM) was introduced as a sacrificial electron donor, promoting the redox cycling of Co^2+^/Co^3+^ and improving radical generation. The solution pH (3–9) was adjusted using 0.1 M HCl or NaOH before PMS addition. All reactions were conducted at ambient temperature (25 ± 2 °C). At predetermined time intervals (30–120 min), 5 mL aliquots were withdrawn, immediately quenched with excess methanol to scavenge residual radicals, and filtered through 0.45 µm syringe filters. The remaining concentrations of NO_3_^−^, NO_2_^−^, and NH_4_^+^ were quantified using ion chromatography (IC; Metrohm 930 Compact IC Flex (Thermo Fisher Scientific Inc., Waltham, MA, USA)). Control experiments were carried out in the absence of PMS, Co–MP, or formic acid to differentiate the relative contributions of adsorption, catalytic activation, and redox-mediated degradation processes. Reproducibility was confirmed by performing at least three independent experiments under identical conditions, yielding a relative standard deviation (RSD) of less than 5%.

### 3.5. RSM Design

RSM was employed to optimize the operational parameters and evaluate their interactive effects on nitrate degradation efficiency in the Co–MP/PMS system. Following preliminary tests that identified the optimal concentrations of PMS and formic acid, five key parameters were selected as independent input variables: reaction time (X_1_), pH (X_2_), initial nitrate concentration (X_3_), microplastic dosage (X_4_), and cobalt concentration (X_5_) (see Supplementary Mterial). A Central Composite Design (CCD) was adopted to systematically explore the experimental domain and develop a predictive quadratic model. Each factor was evaluated at three coded levels (−1, 0, +1), resulting in a total of 33 experimental runs. The nitrate removal efficiency (%) was chosen as the response variable (Y). The design and statistical analyses were performed using Minitab 16 software (Minitab LLC, State College, PA, USA). The experimental data were fitted to a second-order polynomial equation of the form:

(10) $$y=\;b_{0}+\;\sum_{i=1}^{k}b_{i}x_{i}+\sum_{i=1}^{k}b_{ii}x_{ii}^{2}+\;\sum_{i=1}^{k}\sum_{j=i+1}^{k}b_{ij}x_{i}x_{j}+\varepsilon$$

where *y* represents the predicted nitrate removal efficiency (%), $x_{i}$ and $x_{j}$ are the independent variables (factors) included in the experimental design, where i≠j for interaction terms. $b_{0}$ is the model intercept, $b_{i}$ are the linear coefficients, $b_{ii}$ are the quadratic coefficients, and $b_{ij}$ correspond to the interaction coefficients between variables.

Analysis of variance (ANOVA) was applied to assess the statistical significance of the model and its parameters (*p* < 0.05). Diagnostic plots were generated to verify model adequacy, while three-dimensional response surface and contour plots were used to visualize the interaction effects and determine optimal operating conditions. The predicted optimum was subsequently validated through confirmatory experiments.

## 4. Conclusions

This study demonstrates that Co–MP serves as an efficient and durable redox catalyst for formic-acid-assisted denitrification in a PMS-activated system. The Co–MP catalyst, with a confirmed cobalt content of 5.2%, achieved an exceptional nitrate removal efficiency of 90.6% under the RSM-derived optimal conditions: pH 5.5, a catalyst dosage of 1.5 g L^−1^, and a Co(II) concentration of 60 mg L^−1^ over 120 min. The statistical model proved highly reliable, with an R^2^ value of 96.31%, and the experimental result was in excellent agreement with the predicted efficiency of 90.93%. The process facilitated a substantial 86.7% reduction in total nitrogen, confirming that denitrification to gaseous species was the primary removal pathway, with only transient accumulation of nitrite (peak of 3.0 mg L^−1^) and minimal ammonium formation (1.0 mg L^−1^). The transformation followed a stepwise reductive pathway (NO_3_^−^ → NO_2_^−^ → NO → N_2_O → N_2_), mediated by the Co(II)/Co(III) cycle with formic acid acting as the primary electron donor. PMS played an auxiliary role by reoxidizing reduced cobalt species (Co(I) or Co–H) to sustain continuous catalytic turnover. Critically, the catalyst exhibited excellent stability, with cobalt leaching maintained at a very low level of ~0.05 mg L^−1^. By achieving high nitrate degradation and significant total nitrogen removal while valorizing problematic waste material, this research provides a sustainable and innovative solution for advanced water treatment. This study demonstrates a dual-remediation strategy that concurrently addresses nitrate pollution and plastic waste. An important aspect of the dual-remediation strategy is ensuring that the Co–MP catalyst can be effectively recovered after treatment. Owing to the low density of the polyethylene support, the material can be readily separated by flotation or skimming, minimizing the risk of MP discharge. The future incorporation of magnetic components may further enhance recovery efficiency through magnetic separation. Addressing catalyst retrieval and end-of-life management is essential for the sustainable and environmentally responsible application of this technology. The innovation lies in the functional valorization of MPs, which are repurposed as a stable, low-cost support for cobalt in a PMS-activated advanced oxidation process. This approach capitalizes on the inherent durability and surface properties of MPs, transforming them from persistent pollutants into an effective heterogeneous catalyst. Consequently, this method not only purifies water by reducing the nitrate load but also provides a potential end-of-life pathway for plastic debris. This synergistic process embodies a circular economy principle in environmental engineering, converting a waste liability into a functional asset for sustainable water treatment.

## Figures and Tables

**Table 1:** ANOVA for Nitrate Removal Efficiency (%).

Source	DF	Adj SS	Adj MS	F-Value	*p*-Value
Model	20	5209.07	260.45	15.64	0.000
Linear	5	2229.51	445.90	26.78	0.000
pH	1	38.14	38.14	2.29	0.156
Time	1	117.04	117.04	7.03	0.021
C_0_	1	0.53	0.53	0.03	0.861
Cs	1	896.06	896.06	53.81	0.000
Co(II)	1	1177.74	1177.74	70.72	0.000
Square	5	2883.49	576.70	34.63	0.000
pH*pH	1	187.03	187.03	11.23	0.006
Time*Time	1	0.53	0.53	0.03	0.861
C0*C0	1	33.08	33.08	1.99	0.184
Cs*Cs	1	30.42	30.42	1.83	0.201
Co(II)*Co(II)	1	45.85	45.85	2.75	0.123
2-Way Interaction	10	96.07	9.61	0.58	0.804
pH*Time	1	16.81	16.81	1.01	0.335
pH*C0	1	18.49	18.49	1.11	0.313
pH*Cs	1	3.80	3.80	0.23	0.641
pH*Co(II)	1	0.09	0.09	0.01	0.943
Time*C0	1	16.00	16.00	0.96	0.346
Time*Cs	1	1.32	1.32	0.08	0.783
Time*Co(II)	1	6.76	6.76	0.41	0.536
C0*Cs	1	17.22	17.22	1.03	0.329
C0*Co(II)	1	2.25	2.25	0.14	0.720
Cs*Co(II)	1	13.32	13.32	0.80	0.389
Error	12	199.83	16.65		
Lack-of-Fit	6	199.62	33.27	931.54	0.000
Pure Error	6	0.21	0.04		
Total	32	5408.90			

## Data Availability

Data is contained within the article or Supplementary Material.

## Supplementary Materials

The following supporting information can be downloaded at: https://www.mdpi.com/article/10.3390/molecules30234591/s1.
