# Supplementary material for: Cobalt-Immobilized Microplastics as a Functional Catalyst for PMS-Based Nitrate Degradation: Optimization Using Response Surface Methodology
Source: Molecules. 2025 Nov 29;30(23):4591. doi: 10.3390/molecules30234591 (PMC12692958; doi:10.3390/molecules30234591)
Supplement: Supplementary file 1 [file molecules-30-04591-s001.zip › molecules-3976933-supplementary.pdf]

pH	Time	C0	Cs	Co(II)	R (%)
6	75	75	1.0	40	78.5
9	120	50	0.5	60	60.2
6	75	75	1.0	40	78.5
3	120	100	0.5	60	65.8
3	30	50	0.5	60	54.6
9	30	100	0.5	60	52.3
6	75	75	1.0	40	78.0
3	120	50	1.5	60	82.4
3	120	50	0.5	20	46.1
6	75	75	1.0	40	78.5
9	120	100	1.5	60	73.9
3	30	100	1.5	60	69.4
9	120	50	1.5	20	58.7
9	120	100	0.5	20	44.9
6	75	75	1.0	40	78.5
9	30	50	1.5	60	74.1
9	30	50	0.5	20	43.2
6	75	75	1.0	40	78.5
3	30	50	1.5	20	56.8
9	30	100	1.5	20	49.6
3	30	100	0.5	20	42.5
3	120	100	1.5	20	63.9
6	75	50	1.0	40	71.3
6	75	100	1.0	40	82.0
6	75	75	1.0	20	66.7
6	75	75	0.5	40	72.9
6	120	75	1.0	40	76.1
6	30	75	1.0	40	83.6
6	75	75	1.0	60	85.3
9	75	75	1.0	40	70.8
3	75	75	1.0	40	72.4
6	75	75	1.5	40	80.7
6	75	75	1.0	40	78.5
